# Usefulness of seminested polymerase chain reaction for screening blood donors at risk for malaria in Spain.

**DOI:** 10.3201/eid0706.010632

**Published:** 2001

**Authors:** A Benito, J M Rubio


### Usefulness of Seminested Polymerase Chain Reaction for Screening Blood Donors at Risk for Malaria in Spain

**To the Editor:** Assurance of blood safety relies on effective public health surveillance for infectious diseases. Appropriate management of emerging disease threats requires that this surveillance be combined with screening measures to eliminate or minimize the risk of transmitting transfusion-associated disease. Diseases with a long and relatively asymptomatic period (malaria) during which microorganisms are present in the blood are of particular concern for transfusion safety.

Many reports have shown that falciparum malaria can remain in the bloodstream for a long time (even years) as an asymptomatic infection (1). Parasitic contamination of donated blood can occur only if the donor has parasitemia, usually asymptomatic, at the time the blood is collected.

In accordance with legislation of the European Community and the United States, travelers who have visited malaria-endemic areas may be accepted as regular blood donors 6 months after return to the nonendemic area, providing they have been free of unexplained febrile illnesses and have not taken antimalarial drugs (2,3). Immigrants or visitors from endemic areas may donate blood 3 years after they leave the area if they have been asymptomatic in the interim. Donations to be used for the preparation of plasma, plasma components, or derivatives devoid of intact erythrocytes are exempt from these restrictions.

Transfusion-associated cases of malaria have occurred in recent years in the European Community and the United States, although the residual risk of receiving a unit of erythrocyte concentrate contaminated with malaria parasites is very low (estimated at 1 case per 4 million units donated) (4). On the other hand, the potential survival of the organism in an infectious form during blood processing and storage should also be investigated. Several reports concur that all *Plasmodium* species can remain viable in stored blood for at least a week (*P. falciparum* malaria has been transmitted by blood stored for 19 days) (5).

The increase in tourism and migration has caused an increase in imported cases of malaria infections in returned travelers and immigrants from malaria-endemic areas. The number of immigrants from different geographic areas of the world is changing the demographic pattern of European countries, including Spain. Since the early 1990s, the birth rate in the migrant population has attained the same rate as that of the Spanish autochthonous population. This fact has led the migrant population to be incorporated into every layer of society, including the agricultural, construction, and health sectors.

Moreover, the shortage of blood reserves in Spanish hospitals and the increase in immigrants who donate blood indicate a need for sensitive diagnostic techniques capable of detecting parasites in blood. Currently no tests are approved in Spain to screen donated blood for malaria, and careful questioning is essential to identify prospective donors at risk for transmitting malaria.

Experience in our Spanish reference laboratory indicates that new laboratory tests should be implemented to screen high-risk donor blood, especially for malaria and Chagas disease. These tests should be sensitive enough to reject all contaminated donations but specific enough to achieve a strong positive predictive value and minimize unnecessary deferral of otherwise acceptable donors.

We describe the potential use of the seminested multiplex malaria polymerase chain reaction (SNM-PCR) to screen potential blood donors at risk (immigrants from malaria-endemic areas) and reduce the time they are excluded as regular blood donors (3 years). We have reported that the SNM-PCR is capable of detecting 0.004 to 0.04 parasites per microliter of blood (6). In accordance with these data, we addressed the following questions: could SNM-PCR be a useful tool to screen prospective donors at risk? If so, could the 3-year deferral period be shortened for donors whose PCR results are negative?

To evaluate SNM-PCR in potential blood donors at risk, we conducted a study, approved by the Ethical Committee of the National Institute of Health, with blood samples from the Red Cross Blood Center in Madrid (Spain). Throughout 2000, our laboratory received 125 samples (5 $cm^3$ of whole blood in EDTA per sample) from blood donors at risk (56 from Colombia, 50 from Ecuador, 3 from Cameroon, 2 from Peru, 2 from Iran, 2 from China, and 1 each from India, Papua New Guinea, Sri Lanka, Mozambique, Mexico, Senegal, Costa Rica, Brazil, Kenya, and Tanzania). All donors at risk had recently (3 months to 1 year) immigrated to Spain.

Five samples (two from Ecuador, two Colombia, and one India) were positive for *P. falciparum* in two amplifications from two different blood extractions per sample. The five PCR-positive blood specimens were negative by microscopy (7) (we assumed that 400 microscopic fields are equivalent to 1 µL of blood). Our results suggest the following: a) the potential of this population to transmit malaria parasites was confirmed (4% of blood samples were positive), justifying the legislation in force (2,3); and b) SNM-PCR could serve as the reference test for screening donor blood, which could increase the use of these blood donations and consequently shorten the deferral period for blood donors.

**A. Benito and J.M. Rubio**
Malaria Reference Laboratory, Parasitology Department, National Center for Microbiology, National Institute of Health "Carlos III," 28220 Majadahonda, Madrid, Spain

### References

1. Bruce-Chwatt LJ. Transfusion malaria revisited. Trop Dis Bull 1982;79:827-39.
2. European Council. Recommendation on the suitability of blood and plasma donors and the screening of donated blood in the European Community. Official Journal L 203 1998 (June 29, 1998) 98/463/EC: p. 14–26.
3. Standards Committee, American Association of Blood Banks. Donors and donor blood. In: Standards for blood banks and transfusion services. Bethesda (MD): American Association of Blood Banks; 1993. p. 5-6.
4. Guerrero IC, Weniger BG, Schultz MG. Transfusion malaria in the United States, 1972-1981. Ann Intern Med 1983;99:221-6.
5. De Silva M, Contreras M, Barbara J. Two cases of transfusion-transmitted (TTM) in the UK. Transfusion 1988;28:86.
6. Rubio JM, Benito A, Berzosa PJ, Roche J, Puente S, Subirats M, et al. Usefulness of seminested multiplex PCR in surveillance of imported malaria in Spain. J Clin Microbiol 1999;37:3260-4.
7. Barker RH, Banchongaksorn T, Courval JM, Suwonkerd W, Rimwungtragoon K, Wirth DF. A simple method to detect *Plasmodium falciparum* directly from blood samples using the polymerase chain reaction. Am J Trop Med Hyg 1992;46:416-26.

